# Synthesis, crystal structure and Hirshfeld surface analysis of a new copper(II) complex based on diethyl 2,2′-(4*H*-1,2,4-triazole-3,5-di­yl)di­acetate

**DOI:** 10.1107/S2056989024008259

**Published:** 2024-08-30

**Authors:** Oleksandr S. Vynohradov, Oleksandr V. Vashchenko, Dmytro M. Khomenko, Roman O. Doroshchuk, Ilona V. Raspertova, Rostyslav D. Lampeka, Alexandru-Constantin Stoica

**Affiliations:** a Department of Chemistry, Taras Shevchenko National University of Kyiv, Volodymyrska str. 64/13, 01601 Kyiv, Ukraine; b "PetruPoni" Institute of Macromolecular Chemistry, Aleea Gr., Ghica Voda 41A, 700487 Iasi, Romania; Harvard University, USA

**Keywords:** copper, copper(II) complex, crystal structure, 1,2,4-triazole, Hirshfeld surface analysis

## Abstract

The title compound, bis­[μ-2,2′-(4*H*-1,2,4-triazole-3,5-di­yl)di­acetato]­bis­[di­aqua­copper(II)] dihydrate, [Cu_2_(C_6_H_5_N_3_O_4_)_2_(H_2_O)_4_]·2H_2_O, is a dinuclear octa­hedral Cu^II^ triazole-based complex. The central copper atoms are hexa-coordinated by two nitro­gen atoms in the equatorial positions, two equatorial oxygen atoms of two carboxyl­ate substituents in position 3 and 5 of the 1,2,4-triazole ring, and two axial oxygen atoms of two water mol­ecules. Two additional solvent water mol­ecules are linked to the title mol­ecule by O—H⋯N and O⋯H—O hydrogen bonds. The crystal structure is built up from the parallel packing of discrete supra­molecular chains running along the *a*-axis direction. Hirshfeld surface analysis suggests that the most important contributions to the surface contacts are from H⋯O/O⋯H (53.5%), H⋯H (28.1%), O⋯O (6.3%) and H⋯C/C⋯H (6.2%) inter­actions. The crystal studied was twinned by a twofold rotation around [100].

## Abstract 2

The synthesis and crystal structure of a new dinuclear copper(II) complex based on ethyl 2,2′-(1*H*-1,2,4-triazole-3,5-di­yl)-di­acetate is reported. Additionally, the results of a Hirshfeld surface analysis of [Cu_2_(C_6_H_5_N_3_O_4_)_2_(H_2_O)_4_]·2H_2_O are described.

## 1. Chemical context

1,2,4-Triazole-based organic compounds have been widely used as ligands for the synthesis of transition-metal complexes (Haasnoot, 2000 ▸; Aromí *et al.*, 2011 ▸; Farooq, 2021 ▸). Depending on the substituents on the azole core, the title ligands can coordinate not only in a monodentate manner (Cudziło *et al.*, 2011 ▸; Zaleski *et al.*, 2005 ▸), but also as a linker binding two metal ions (Drabent *et al.*, 2001 ▸; Zhang *et al.*, 2005 ▸) and thus play an important role in the design of new polynuclear coordination compounds. In particular, copper(II) coordin­ation compounds based on 1,2,4-triazoles have attracted the inter­est of chemists due to their magnetic properties (Petrenko *et al.*, 2020 ▸; Kaase *et al.*, 2014 ▸), bioactivity (Hernández-Gil *et al.*, 2013 ▸; Ferrer *et al.*, 2004 ▸) and catalysis (Thorseth *et al.*, 2013 ▸; Li *et al.*, 2015 ▸). Dinuclear copper(II) complexes can promote single- and double-strand DNA cleavage in both aerobic and anaerobic conditions (Li *et al.*, 2010 ▸). Being much cheaper than most metals, copper(II) coordination compounds are promising substances for exploration as catalysts. Previously we reported that a dinuclear Cu^II^ complex based on 5-methyl-3-(2-pyrid­yl)-1,2,4-triazole as a ligand can selectively catalyse the oxidation of styrene towards benzaldehyde and of cyclo­hexane to KA oil (a mixture of cyclo­hexa­nol and cyclo­hexa­none; Petrenko *et al.*, 2021 ▸). Finally, Cu^II^ complexes can exhibit urease inhibitory activities (Xu *et al.*, 2015 ▸). Since dinuclear copper(II) complexes with triazole bridges can exhibit catalytic properties, we decided to continue our research in this direction. Herein, we describe the synthesis, crystal structure, and results of Hirshfeld surface analysis of the title compound, [Cu_2_(C_6_H_5_N_3_O_4_)_2_(H_2_O)_4_]·2H_2_O, which potentially exhibits catalytic, inhibitory, and magnetic properties.
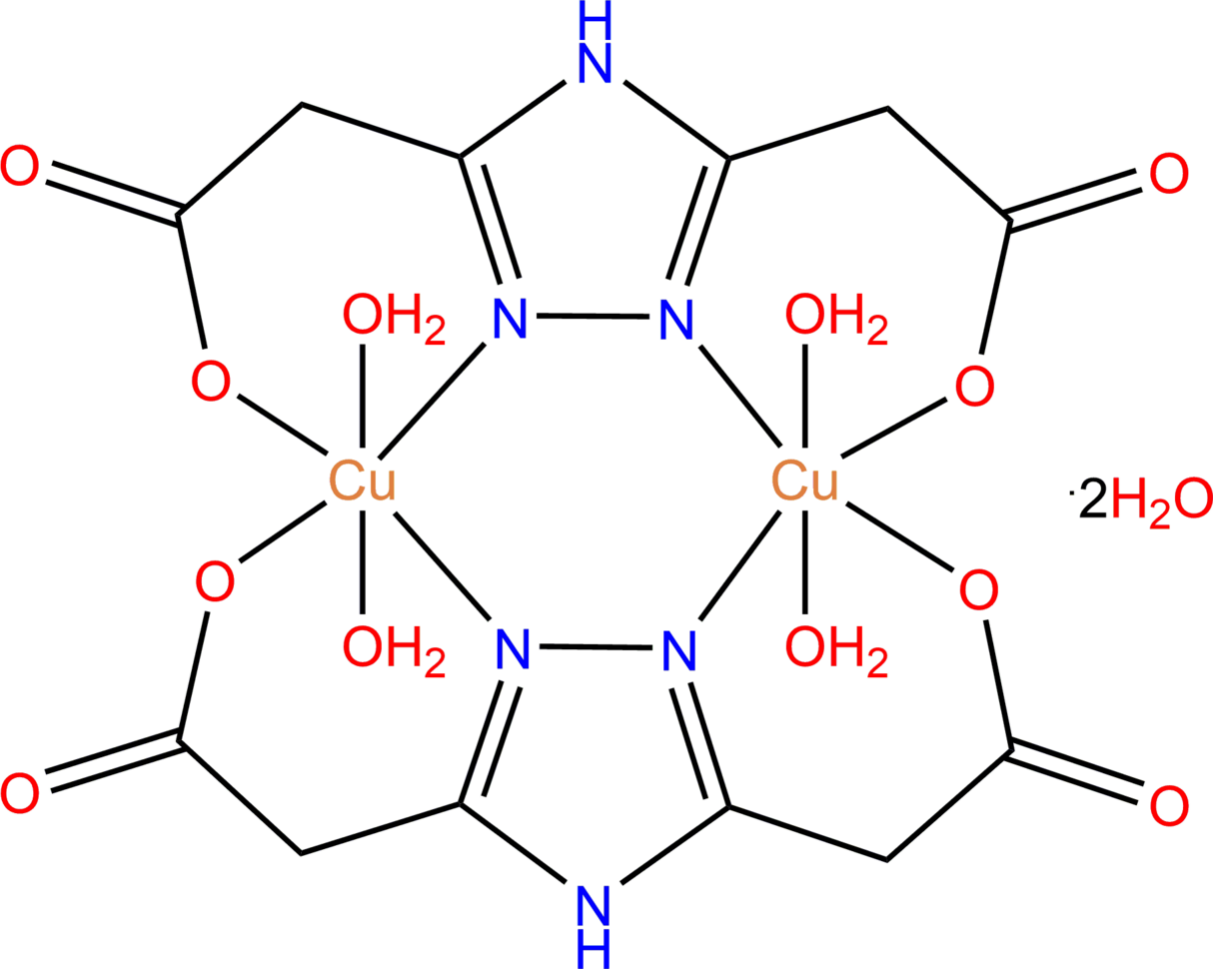


## 2. Structural commentary

**Figure 1:**
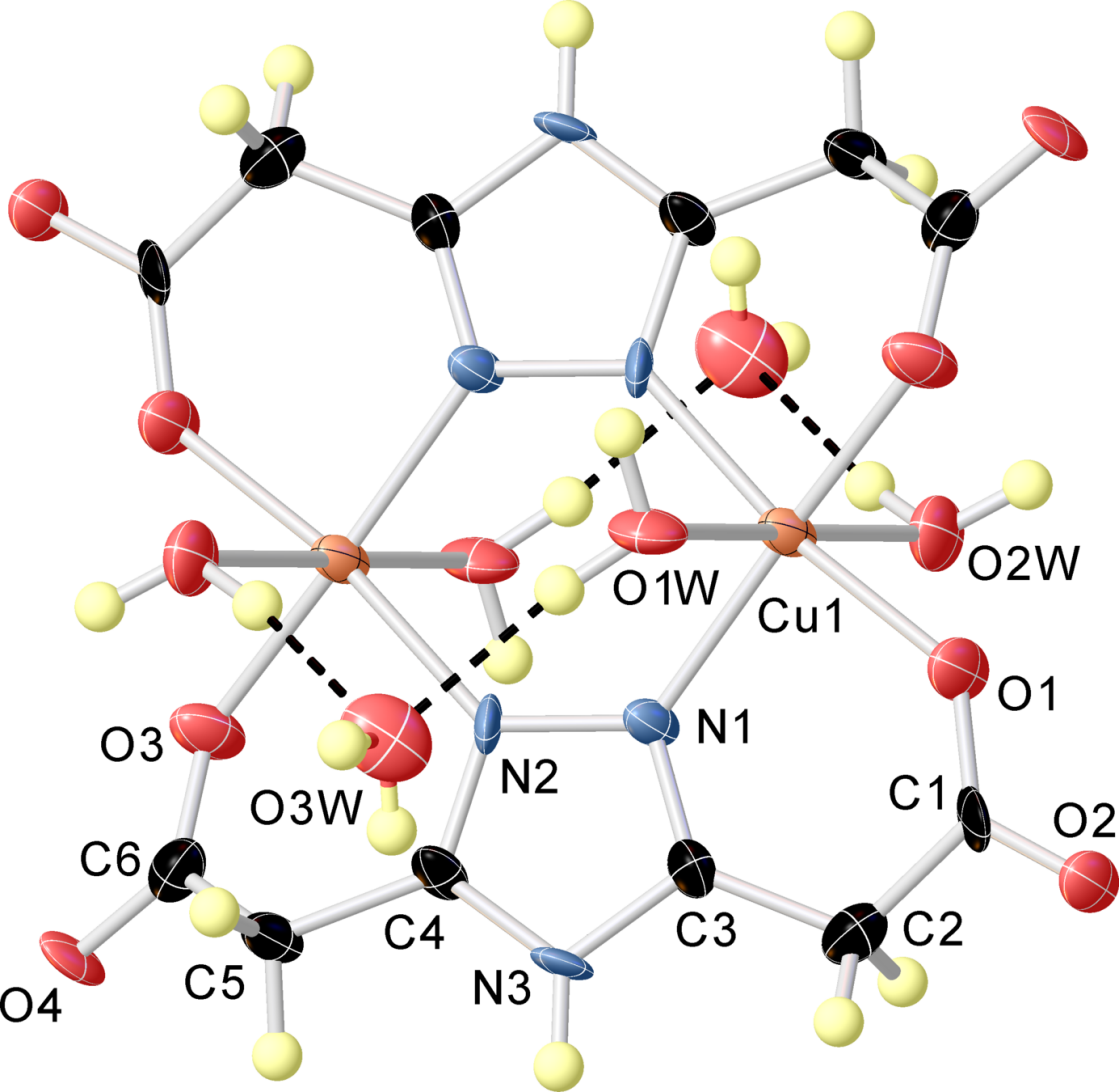
The mol­ecular structure of the title compound with the atom labelling. Displacement ellipsoids are drawn at the 50% probability level.

The title compound (Fig. 1 ▸), a dinuclear copper(II) 1,2,4-triazole-based complex, crystallizes in the monoclinic, *P*2_1_/*n* space group. The asymmetric unit consists of one copper(II) ion, one 4*H*-1,2,4-triazole-3,5-di­carboxyl­ate ligand, two coordinated water mol­ecules and one solvent water mol­ecule. The structure of the title compound can be described as a neutral complex of formula [Cu_2_(C_6_H_5_N_3_O_4_)_2_(H_2_O)_4_]·2H_2_O in which the triazole ligand is coordinated in a tetra­dentate way. The Cu^II^ ion has a distorted N_2_O_4_ octa­hedral geometry formed by two nitro­gen atoms in the equatorial positions with Cu1—N1 = 1.982 (3) Å and Cu1—N2^i^ [symmetry code: (i) −*x* + 1, −*y* + 1, −*z* + 1)] = 1.990 (4) Å bond distances, two equatorial oxygen atoms of two carboxyl­ate substituents in position 3 and 5 of the triazole ring [Cu1—O1 = 1.962 (3) Å and Cu1—O3^i^ = 1.974 (3) Å], and two axial oxygen atoms of two water mol­ecules with Cu1—O1*W* = 2.497 (3) Å and Cu1—O2*W* = 2.484 (3) Å bond distances. The Cu1—Cu1^i^ inter­metallic distance in the complex mol­ecule is 3.9866 (15) Å. Two copper atoms bridged by two 4*H*-1,2,4-triazole-3,5-di­carboxyl­ate ligands form a non-planar six-membered bimetallic ring. In addition, four six-membered non-planar chelate rings are formed due to the presence of carboxyl­ate substituents at the 3 and 5 positions of the 1,2,4-triazole rings. There are medium strength inter­molecular O—H⋯O hydrogen bonds between the main compound and solvent water mol­ecules. Inter­molecular N—H⋯O and C—H⋯O hydrogen bonds between two complex mol­ecules are also observed (Table 1 ▸).

## 3. Supra­molecular features

**Figure 2:**
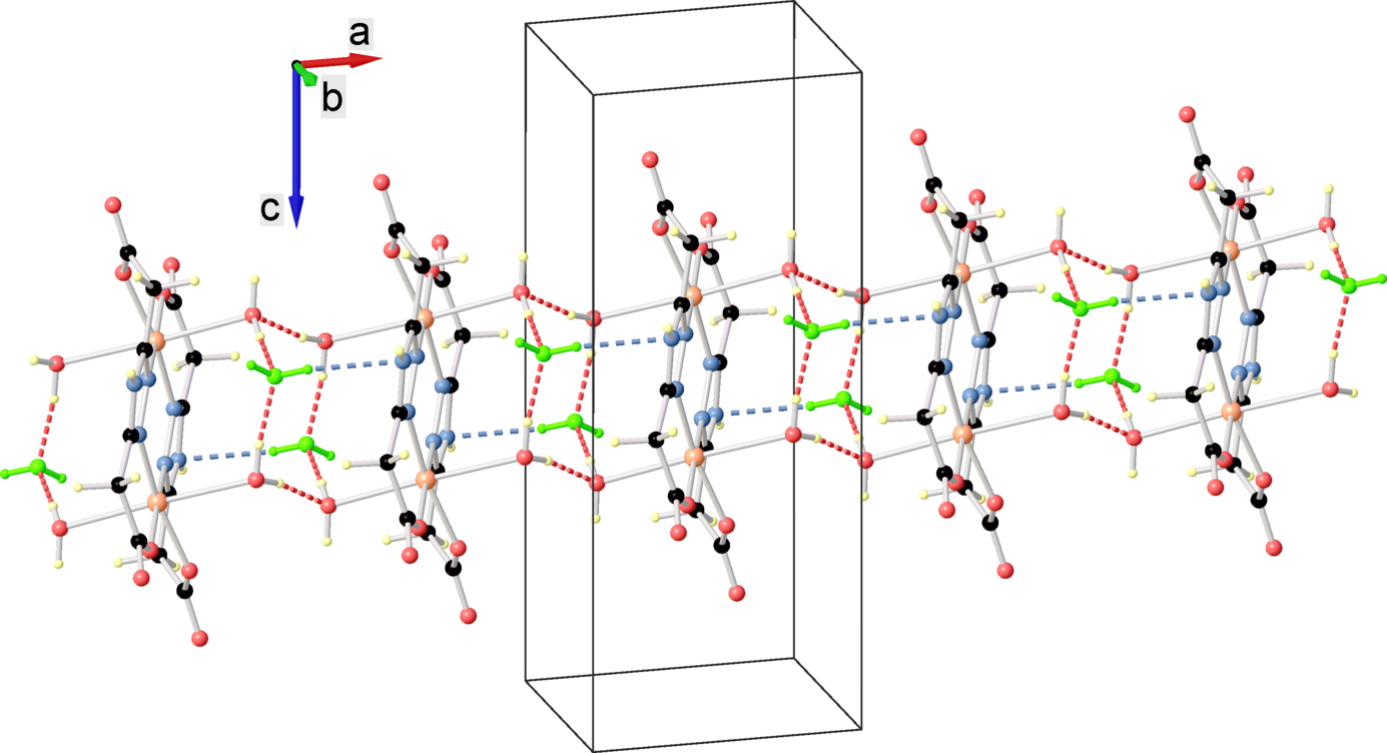
One-dimensional supra­molecular chain running parallel to the *a* axis and viewed along the *b* axis. Solvent water mol­ecules are shown in green, O—H⋯O and O—H⋯N hydrogen bonds are shown as red and blue dotted lines, respectively.

**Figure 3:**
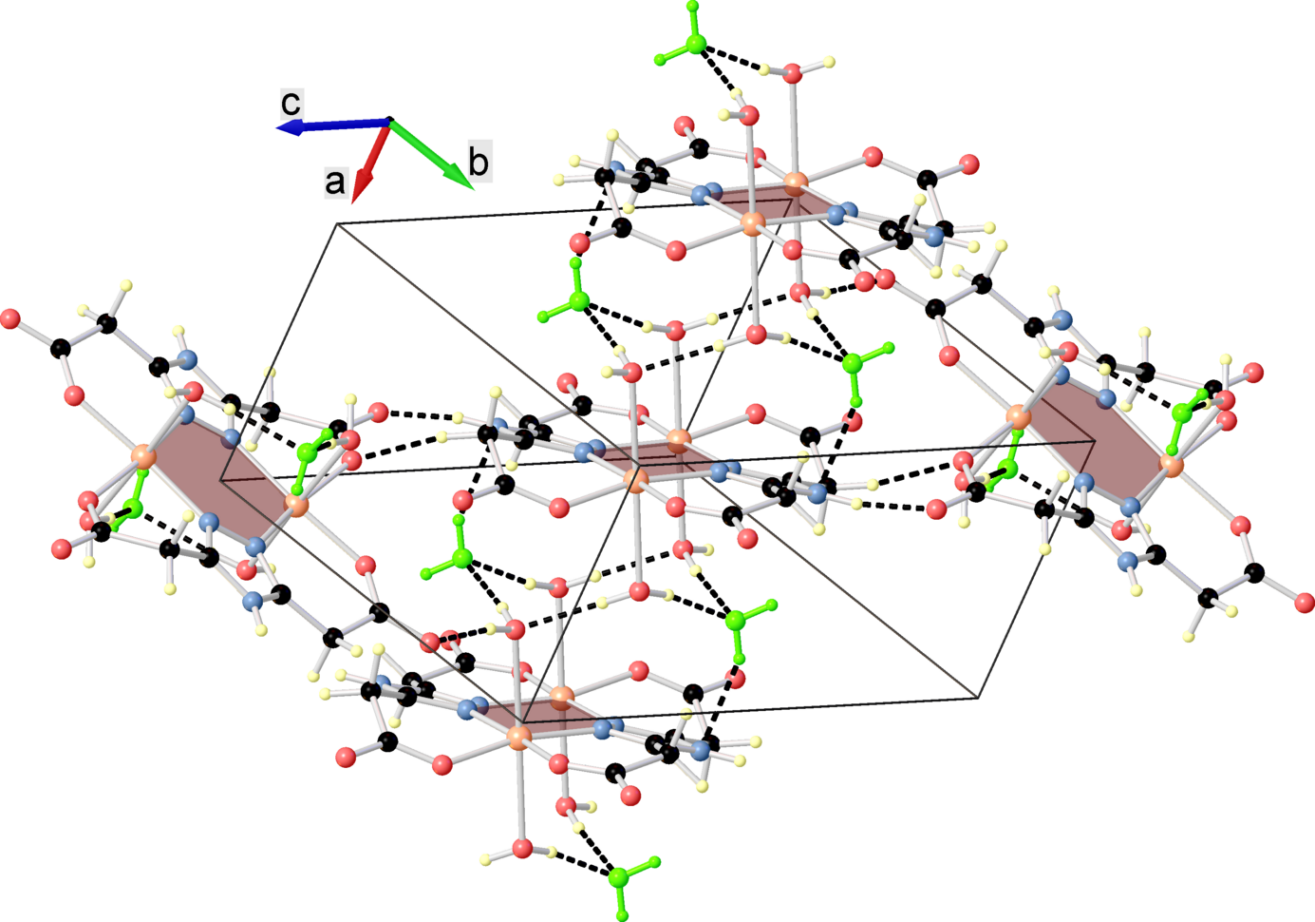
Partial view of the crystal packing showing hydrogen-bond contacts between adjacent mol­ecules.

The crystal structure is built up from the parallel packing of discrete supra­molecular chains running along the *a*-axis direction with a Cu⋯Cu separation of 6.5248 (11) Å (Fig. 2 ▸). Within the chain, the complex mol­ecules inter­act through O—H⋯O hydrogen bonds, while the association with the inter­stitial water mol­ecules occurs *via* O—H⋯O and N—H⋯O hydrogen bonds (Fig. 3 ▸, Table 1 ▸).

## 4. Database survey

A search of the Cambridge Structural Database (CSD version 5.41, update of November 2021; Groom *et al.*, 2016 ▸) for the Cu_2_(C_2_N_3_)_2_O_4_ moiety (two 1,2,4-triazole ring skeletons connected *via* two Cu atoms; each copper atom is coordinated by two oxygen atoms) revealed 48 hits. Most similar to the title compound are dinuclear copper(II) complexes with following database refcodes: COCZAV (Ferrer *et al.*, 1999 ▸), DODRET and DODRIX (Prins *et al.*, 1985 ▸), FIVGEY (Matthews *et al.*, 2003 ▸), JOZXAX (van Koningsbruggen *et al.*, 1992 ▸) and VALZOA (Doroschuk, 2016 ▸). All coordination compounds have many common characteristics, but there are also some minor differences between them. All these dinuclear copper(II) complexes contain two 1,2,4-triazole-based ligands. The triazole derivatives have two substituents at positions 3 and 5 of the triazole ring. The substituents containing donor atoms also participate in coordination with the copper atom. These ligands exhibit bridging functions and link two copper atoms at distances in the range of 3.85 to 4.09 Å. Two six-coordinated copper atoms are involved in the formation of a six-membered ring. There are two water mol­ecules in the axial positions of the central copper atom in the title compound and the compound JOZXAX. In other complexes, one axial position in the geometric environment of the copper atom is occupied by a water mol­ecule, while the second axial position is typically occupied by an anion of an inorganic salt. The title compound crystallizes in the monoclinic *P*2_1_/*n* space group. Five complexes crystallized in the triclinic, *P*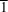 space group, while JOZXAX crystallized in the monoclinic *C*2/*c* space group.

## 5. Hirshfeld surface analysis

**Figure 4:**
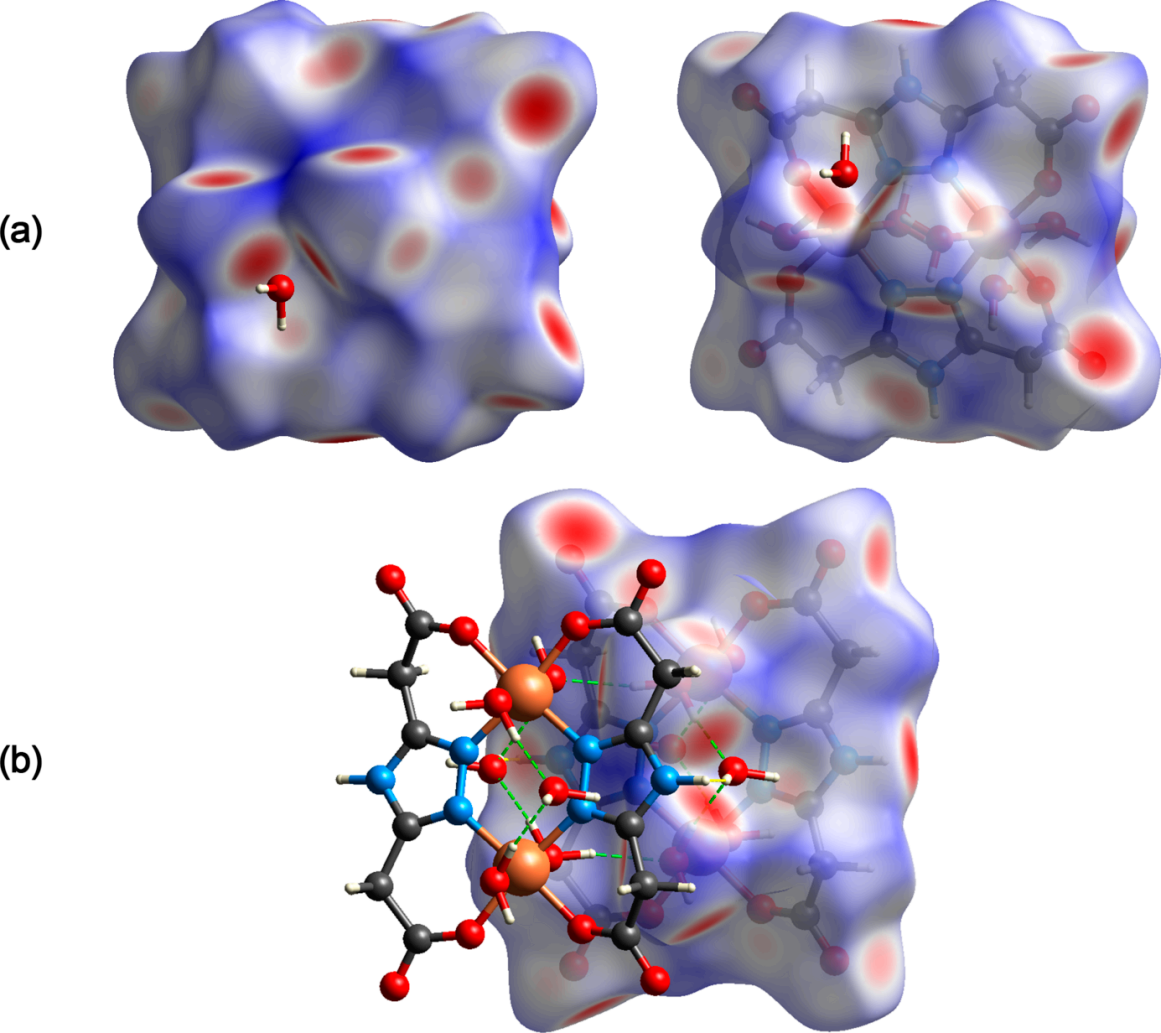
(*a*) Two projections of the Hirshfeld surfaces mapped over *d*_norm_ showing the inter­molecular inter­actions within the mol­ecule and (*b*) an illustration of selected O—H⋯O and O—H⋯N inter­actions depicted by green and yellow dashed lines, respectively.

**Figure 6:**
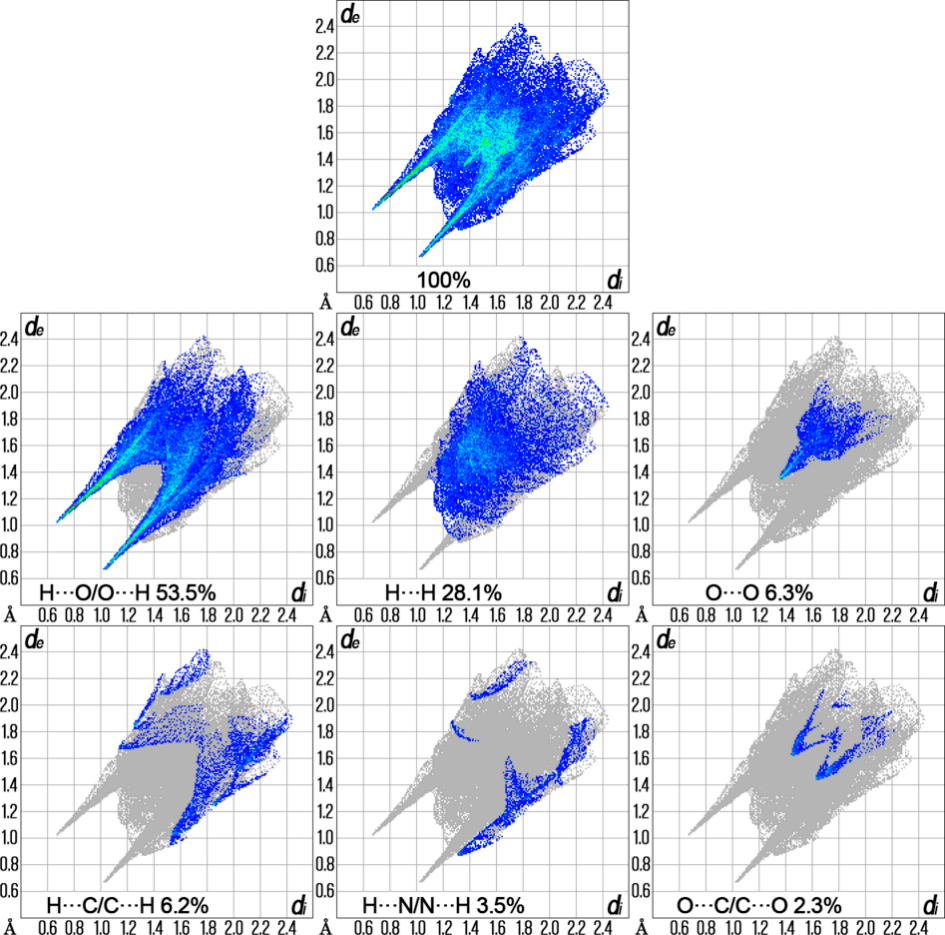
The overall two-dimensional fingerprint plot and those delineated into specified inter­actions.

The Hirshfeld surface analysis was performed and the associated two-dimensional fingerprint plots were generated using *Crystal Explorer 17.5* software (Spackman *et al.*, 2021 ▸), with a standard resolution of the three-dimensional *d*_norm_ surfaces (shown in Figs. 4 ▸ and 5 ▸). The dark-red spots arise as a result of short inter­atomic contacts and represent negative *d*_norm_ values on the surface, while the other weaker inter­molecular inter­actions appear as light-red spots. The Hirshfeld surfaces mapped over *d*_norm_ are shown for the H⋯O/O⋯H, H⋯H, O⋯O, H⋯C/C⋯H, H⋯N/N⋯H and O⋯C/C⋯O contacts (Fig. 6 ▸), the overall two-dimensional fingerprint plot and the decomposed two-dimensional fingerprint plots are given in Fig. 7. Two pairs of N3—H3⋯O4 inter­atomic contacts with lengths of 1.696 Å are the shortest. The most significant contributions to the overall crystal packing are from H⋯O/O⋯H (53.5%), H⋯H (28.1%), O⋯O (6.3%) and H⋯C/C⋯H (6.2%) contacts. The predominance of contributions from H⋯H and H⋯O contacts to the overall crystal packing is typical not only for the title compound and other dinuclear copper(II) complexes with triazole-containing ligands but also of copper(II) coordination compounds with azole-based ligands in general. There is a small contribution from H⋯N/N⋯H (3.5%) and O⋯C/C⋯O (2.3%) weak inter­molecular contacts. The relative percentage contributions to the overall Hirshfeld surface by elements: H⋯all atoms = 55.4%, O⋯all atoms = 35.6%, C⋯all atoms = 6.1%, N⋯all atoms = 3.0% and Cu⋯all atoms = 0%. In addition, qu­anti­tative physical properties of the Hirshfeld surface for this compound were obtained, such as mol­ecular volume (444.48 Å^3^), surface area (384.06 Å^2^), globularity (0.733) and asphericity (0.063). The asphericity value for the title compound at 0.063 is close to zero, indicating a near isotropic nature. The globularity value (0.733) is less than one, suggesting a modest deviation from a spherical surface and indicating that this mol­ecular surface is more structured compared to a sphere.

## 6. Synthesis and crystallization

**[Cu_2_(H*****L*****)_2_(H_2_O)_4_]·2H_2_O.** An aqueous solution (2 ml) of Cu(NO_3_)_2_**·**6H_2_O (0.296 g, 1 mmol) was added to 2 ml of an aqueous solution of ethyl 2,2′-(1*H*-1,2,4-triazole-3,5-di­yl)di­acetate (0.241 g, 1 mmol) to give a clear blue solution. The blue crystals that precipitated after 2 days were filtered off, washed with water, and dried in air (Kiseleva *et al.*, 1990 ▸). Yield 0.247 g (82.18%). IR data (in KBr, cm^−1^): 3404, 3224, 1638, 1620, 1606, 1568, 1540, 1454, 1418, 1400, 1386, 1274, 1250, 1052, 956, 752, 644, 578. Analysis calculated for C_12_H_22_Cu_2_N_6_O_14_ (601.43): C, 23.96%; H, 3.69%; N, 13.97%. Found: C, 23.88%; H, 3.72%; N, 13.88%.

## 7. Refinement

Crystal data, data collection and structure refinement details are summarized in Table 2 ▸. The crystal studied was twinned by a twofold rotation around [100]. The corresponding HKLF5 generated by the *CrysAlis* program was used for refinement. The O- and N-bound hydrogen atoms were identified in difference-Fourier maps and refined isotropically with positional restraints. All other H atoms were placed in calculated positions and refined using a riding model with C—H = 0.99 Å and *U*_iso_(H) = 1.2*U*_eq_(C).

## Supplementary Material

Crystal structure: contains datablock(s) I. DOI: 10.1107/S2056989024008259/oi2010sup1.cif

Structure factors: contains datablock(s) I. DOI: 10.1107/S2056989024008259/oi2010Isup2.hkl

Supporting information file. DOI: 10.1107/S2056989024008259/oi2010Isup3.cdx

FT-IR spectrum of the title compound. DOI: 10.1107/S2056989024008259/oi2010sup4.jpg

Powder X-ray diffraction pattern of the title compound. DOI: 10.1107/S2056989024008259/oi2010sup5.jpg

CCDC reference: 2378887

Additional supporting information:  crystallographic information; 3D view; checkCIF report

## Figures and Tables

**Figure 5:**
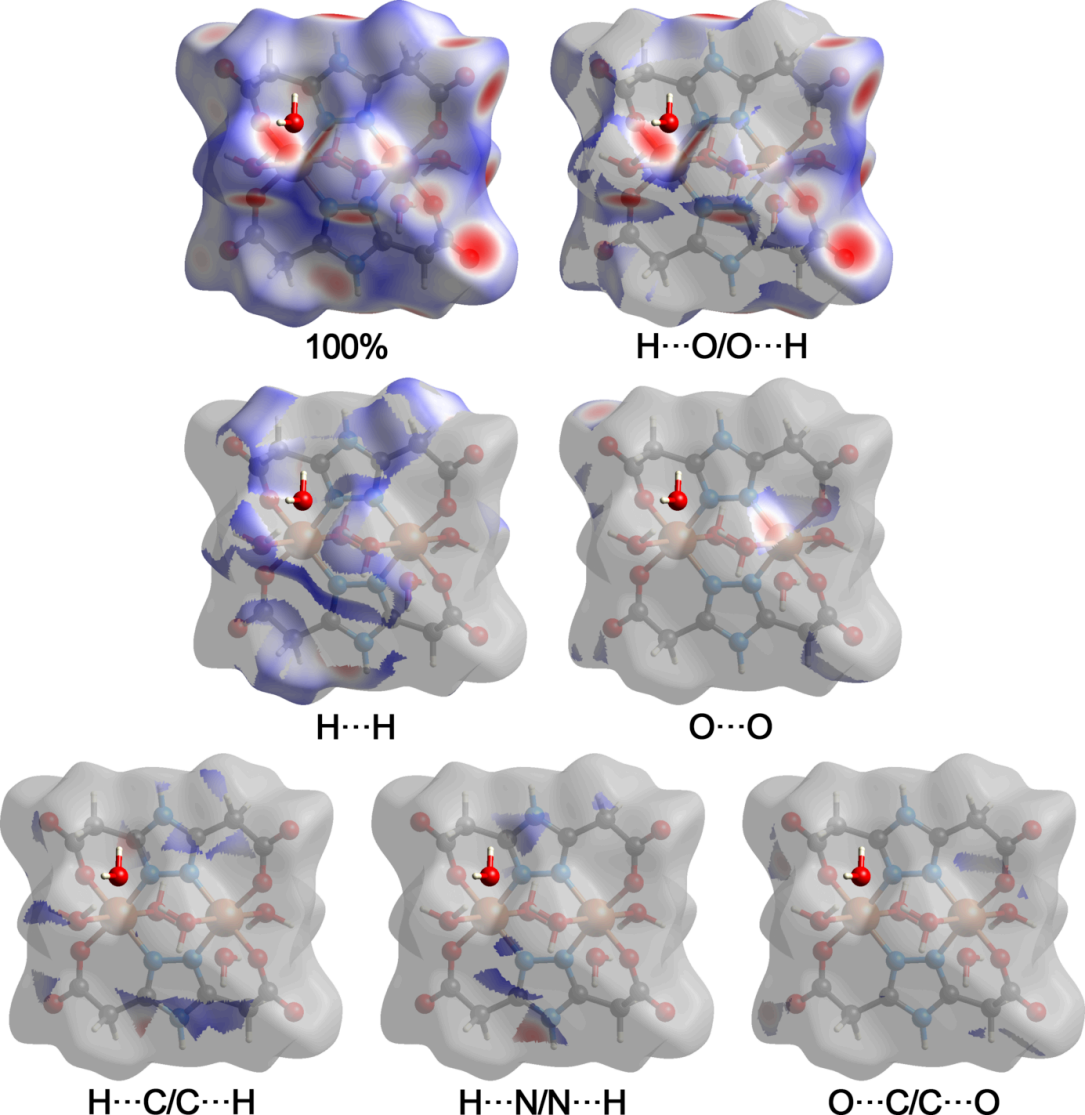
Hirshfeld surface representations with the function *d*_norm_ plotted onto the surface for the different inter­actions.

**Table 1:** Hydrogen-bond geometry (Å, °)

*D*—H⋯*A*	*D*—H	H⋯*A*	*D*⋯*A*	*D*—H⋯*A*
O1*W*—H1*WA*⋯O4^i^	0.87	1.91	2.762 (5)	165
O1*W*—H1*WB*⋯O3*W*	0.87	1.94	2.797 (5)	169
O2*W*—H2*WA*⋯O1*W*^ii^	0.85	2.03	2.880 (4)	170
O2*W*—H2*WB*⋯O2^iii^	0.87	1.89	2.744 (4)	167
N3—H3⋯O4^iv^	0.88	1.81	2.670 (5)	165
C5—H5*B*⋯O3^iv^	0.99	2.32	3.257 (6)	158

Symmetry codes: (i) 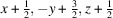; (ii) 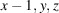; (iii) 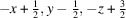; (iv) 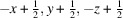.

**Table 2:** Experimental details

Crystal data	
Chemical formula	[Cu_2_(C_6_H_5_N_3_O_4_)_2_(H_2_O)_4_]·2H_2_O
*M*_r_	601.44
Crystal system, space group	Monoclinic, *P*2_1_/*n*
Temperature (K)	200
*a*, *b*, *c* (Å)	6.5176 (13), 9.4854 (19), 15.967 (2)
β (°)	93.035 (15)
*V* (Å^3^)	985.7 (3)
*Z*	2
Radiation type	Mo *K*α
μ (mm^−1^)	2.25
Crystal size (mm)	0.56 × 0.37 × 0.33 × 0.22 (radius)

Data collection	
Diffractometer	Xcalibur, Eos
Absorption correction	For a sphere (*CrysAlis PRO*; Rigaku OD, 2024 ▸)
*T*_min_, *T*_max_	0.488, 0.498
No. of measured, independent and observed [*I* > 2σ(*I*)] reflections	2569, 2569, 1969
*R*_int_	0.053
(sin θ/λ)_max_ (Å^−1^)	0.686

Refinement	
*R*[*F*^2^ > 2σ(*F*^2^)], *wR*(*F*^2^), *S*	0.041, 0.096, 1.00
No. of reflections	2569
No. of parameters	159
No. of restraints	18
H-atom treatment	H-atom parameters constrained
Δρ_max_, Δρ_min_ (e Å^−3^)	0.63, −0.53

Computer programs: *CrysAlis PRO* (Rigaku OD, 2024 ▸), *SHELXS* (Sheldrick, 2008 ▸), *SHELXL2018/3* (Sheldrick, 2015 ▸) and *OLEX2* (Dolomanov *et al.*, 2009 ▸).

## supplementary crystallographic information

### Bis[µ-2,2'-(4H-1,2,4-triazole-3,5-diyl)diacetato]bis[diaquacopper(II)] dihydrate Crystal data

**Table:**

[Cu_2_(C_6_H_5_N_3_O_4_)_2_(H_2_O)_4_]·2H_2_O	*F*(000) = 612
*M**_r_* = 601.44	*D*_x_ = 2.026 Mg m^−^^3^
Monoclinic, *P*2_1_/*n*	Mo *K*α radiation, λ = 0.71073 Å
*a* = 6.5176 (13) Å	Cell parameters from 833 reflections
*b* = 9.4854 (19) Å	θ = 2.5–28.3°
*c* = 15.967 (2) Å	µ = 2.25 mm^−^^1^
β = 93.035 (15)°	*T* = 200 K
*V* = 985.7 (3) Å^3^	Prism, clear intense green
*Z* = 2	0.56 × 0.37 × 0.33 × 0.22 (radius) mm

### Bis[µ-2,2'-(4H-1,2,4-triazole-3,5-diyl)diacetato]bis[diaquacopper(II)] dihydrate Data collection

**Table:**

Xcalibur, Eos diffractometer	1969 reflections with *I* > 2σ(*I*)
Detector resolution: 16.1593 pixels mm^-1^	*R*_int_ = 0.053
ω scans	θ_max_ = 29.2°, θ_min_ = 2.5°
Absorption correction: for a sphere (CrysAlisPro; Rigaku OD, 2024)	*h* = −8→8
*T*_min_ = 0.488, *T*_max_ = 0.498	*k* = −12→12
2569 measured reflections	*l* = −21→21
2569 independent reflections	

### Bis[µ-2,2'-(4H-1,2,4-triazole-3,5-diyl)diacetato]bis[diaquacopper(II)] dihydrate Refinement

**Table:**

Refinement on *F*^2^	Primary atom site location: structure-invariant direct methods
Least-squares matrix: full	Hydrogen site location: mixed
*R*[*F*^2^ > 2σ(*F*^2^)] = 0.041	H-atom parameters constrained
*wR*(*F*^2^) = 0.096	*w* = 1/[σ^2^(*F*_o_^2^) + (0.0483*P*)^2^] where *P* = (*F*_o_^2^ + 2*F*_c_^2^)/3
*S* = 1.00	(Δ/σ)_max_ = 0.001
2569 reflections	Δρ_max_ = 0.63 e Å^−^^3^
159 parameters	Δρ_min_ = −0.53 e Å^−^^3^
18 restraints	

### Bis[µ-2,2'-(4H-1,2,4-triazole-3,5-diyl)diacetato]bis[diaquacopper(II)] dihydrate Special details

**Table:**

Geometry. All esds (except the esd in the dihedral angle between two l.s. planes) are estimated using the full covariance matrix. The cell esds are taken into account individually in the estimation of esds in distances, angles and torsion angles; correlations between esds in cell parameters are only used when they are defined by crystal symmetry. An approximate (isotropic) treatment of cell esds is used for estimating esds involving l.s. planes.
Refinement. Refined as a 2-component twin.

### Bis[µ-2,2'-(4H-1,2,4-triazole-3,5-diyl)diacetato]bis[diaquacopper(II)] dihydrate Fractional atomic coordinates and isotropic or equivalent isotropic displacement parameters (Å^2^)

**Table:**

	*x*	*y*	*z*	*U*_iso_*/*U*_eq_	
Cu1	0.48120 (9)	0.59062 (7)	0.61179 (3)	0.01441 (17)	
O1	0.4081 (5)	0.7706 (4)	0.66289 (18)	0.0196 (8)	
O1W	0.8274 (4)	0.6812 (4)	0.57854 (18)	0.0176 (8)	
H1WA	0.849334	0.760096	0.605348	0.026*	
H1WB	0.826653	0.704497	0.525777	0.026*	
O2	0.3198 (5)	0.9950 (4)	0.67791 (19)	0.0167 (8)	
O2W	0.1424 (5)	0.4979 (4)	0.65012 (18)	0.0220 (8)	
H2WA	0.058325	0.553892	0.624170	0.033*	
H2WB	0.134335	0.500812	0.704360	0.033*	
O3	0.3906 (5)	0.4446 (3)	0.27539 (18)	0.0181 (8)	
O3W	0.8739 (6)	0.7755 (4)	0.4147 (2)	0.0255 (9)	
H3WA	0.888191	0.700243	0.386753	0.038*	
H3WB	0.812698	0.836952	0.383823	0.038*	
O4	0.3227 (4)	0.5597 (3)	0.15734 (18)	0.0148 (8)	
N1	0.3931 (5)	0.6605 (4)	0.4985 (2)	0.0110 (9)	
N2	0.4400 (5)	0.6031 (4)	0.42206 (19)	0.0104 (8)	
N3	0.3370 (5)	0.8181 (4)	0.4021 (2)	0.0115 (9)	
H3	0.302775	0.898014	0.377062	0.014*	
C1	0.3396 (6)	0.8877 (6)	0.6350 (3)	0.0114 (11)	
C2	0.2603 (7)	0.8982 (5)	0.5444 (3)	0.0190 (12)	
H2A	0.108417	0.894494	0.543222	0.023*	
H2B	0.298467	0.992262	0.523253	0.023*	
C3	0.3309 (6)	0.7904 (6)	0.4839 (3)	0.0112 (11)	
C4	0.4041 (6)	0.7029 (5)	0.3657 (3)	0.0126 (11)	
C5	0.4403 (7)	0.6970 (5)	0.2737 (3)	0.0134 (11)	
H5A	0.587709	0.714517	0.265692	0.016*	
H5B	0.360820	0.773275	0.244772	0.016*	
C6	0.3793 (6)	0.5556 (6)	0.2329 (3)	0.0145 (12)	

### Bis[µ-2,2'-(4H-1,2,4-triazole-3,5-diyl)diacetato]bis[diaquacopper(II)] dihydrate Atomic displacement parameters (Å^2^)

**Table:**

	*U*^11^	*U*^22^	*U*^33^	*U*^12^	*U*^13^	*U*^23^
Cu1	0.0258 (3)	0.0090 (3)	0.0082 (2)	0.0024 (3)	−0.0025 (2)	0.0000 (3)
O1	0.0343 (19)	0.0125 (18)	0.0119 (15)	0.0043 (18)	0.0005 (15)	−0.0056 (15)
O1W	0.0274 (17)	0.013 (2)	0.0122 (15)	−0.0017 (18)	−0.0028 (13)	−0.0004 (17)
O2	0.0299 (18)	0.0093 (19)	0.0107 (16)	0.0032 (18)	0.0007 (14)	−0.0018 (15)
O2W	0.0337 (19)	0.019 (2)	0.0129 (16)	0.012 (2)	−0.0023 (14)	0.0013 (17)
O3	0.034 (2)	0.008 (2)	0.0124 (16)	−0.0008 (17)	−0.0041 (14)	0.0030 (15)
O3W	0.038 (2)	0.017 (2)	0.0212 (17)	0.000 (2)	−0.0018 (16)	−0.0045 (19)
O4	0.0280 (17)	0.010 (2)	0.0061 (15)	−0.0003 (16)	−0.0051 (12)	0.0013 (15)
N1	0.0183 (19)	0.008 (2)	0.0063 (17)	−0.0002 (19)	−0.0012 (15)	0.0007 (17)
N2	0.0103 (16)	0.015 (2)	0.0066 (14)	0.003 (2)	0.0029 (14)	−0.0029 (19)
N3	0.019 (2)	0.008 (2)	0.0077 (18)	0.0014 (19)	−0.0009 (15)	0.0053 (18)
C1	0.013 (2)	0.017 (3)	0.0041 (19)	−0.003 (2)	0.0056 (16)	0.000 (2)
C2	0.026 (3)	0.013 (3)	0.018 (2)	0.005 (3)	0.0021 (18)	−0.001 (3)
C3	0.014 (2)	0.012 (3)	0.007 (2)	0.000 (2)	−0.0001 (17)	0.004 (2)
C4	0.014 (2)	0.013 (3)	0.012 (2)	−0.003 (2)	0.0015 (18)	0.003 (2)
C5	0.019 (2)	0.010 (3)	0.011 (2)	0.001 (2)	−0.0012 (19)	−0.001 (2)
C6	0.011 (2)	0.018 (3)	0.016 (2)	0.004 (2)	0.0031 (17)	−0.001 (2)

### Bis[µ-2,2'-(4H-1,2,4-triazole-3,5-diyl)diacetato]bis[diaquacopper(II)] dihydrate Geometric parameters (Å, º)

**Table:**

Cu1—O1	1.962 (3)	O4—C6	1.243 (5)
Cu1—O1W	2.497 (3)	N1—N2	1.385 (4)
Cu1—O2W	2.484 (3)	N1—C3	1.314 (6)
Cu1—O3^i^	1.974 (3)	N2—C4	1.319 (5)
Cu1—N1	1.982 (3)	N3—H3	0.8800
Cu1—N2^i^	1.990 (4)	N3—C3	1.336 (5)
O1—C1	1.269 (5)	N3—C4	1.323 (6)
O1W—H1WA	0.8703	C1—C2	1.514 (6)
O1W—H1WB	0.8707	C2—H2A	0.9900
O2—C1	1.238 (5)	C2—H2B	0.9900
O2W—H2WA	0.8546	C2—C3	1.495 (6)
O2W—H2WB	0.8709	C4—C5	1.502 (5)
O3—C6	1.253 (5)	C5—H5A	0.9900
O3W—H3WA	0.8491	C5—H5B	0.9900
O3W—H3WB	0.8489	C5—C6	1.534 (6)
			
O1—Cu1—O1W	91.79 (13)	C4—N2—N1	106.2 (4)
O1—Cu1—O2W	88.11 (12)	C3—N3—H3	126.4
O1—Cu1—O3^i^	82.43 (13)	C4—N3—H3	126.4
O1—Cu1—N1	91.35 (14)	C4—N3—C3	107.1 (4)
O1—Cu1—N2^i^	171.18 (13)	O1—C1—C2	119.3 (4)
O2W—Cu1—O1W	177.86 (9)	O2—C1—O1	124.8 (4)
O3^i^—Cu1—O1W	84.77 (12)	O2—C1—C2	115.8 (5)
O3^i^—Cu1—O2W	93.10 (12)	C1—C2—H2A	107.8
O3^i^—Cu1—N1	167.83 (15)	C1—C2—H2B	107.8
O3^i^—Cu1—N2^i^	89.34 (13)	H2A—C2—H2B	107.1
N1—Cu1—O1W	84.98 (13)	C3—C2—C1	118.1 (4)
N1—Cu1—O2W	97.16 (13)	C3—C2—H2A	107.8
N1—Cu1—N2^i^	97.31 (14)	C3—C2—H2B	107.8
N2^i^—Cu1—O1W	90.61 (13)	N1—C3—N3	109.5 (4)
N2^i^—Cu1—O2W	89.17 (13)	N1—C3—C2	129.0 (4)
C1—O1—Cu1	134.8 (3)	N3—C3—C2	121.5 (4)
Cu1—O1W—H1WA	108.5	N2—C4—N3	110.2 (4)
Cu1—O1W—H1WB	109.5	N2—C4—C5	127.5 (4)
H1WA—O1W—H1WB	104.5	N3—C4—C5	122.2 (4)
Cu1—O2W—H2WA	102.5	C4—C5—H5A	108.9
Cu1—O2W—H2WB	109.6	C4—C5—H5B	108.9
H2WA—O2W—H2WB	113.0	C4—C5—C6	113.5 (4)
C6—O3—Cu1^i^	130.3 (3)	H5A—C5—H5B	107.7
H3WA—O3W—H3WB	109.5	C6—C5—H5A	108.9
N2—N1—Cu1	127.3 (3)	C6—C5—H5B	108.9
C3—N1—Cu1	123.1 (3)	O3—C6—C5	119.8 (4)
C3—N1—N2	107.0 (4)	O4—C6—O3	123.9 (5)
N1—N2—Cu1^i^	132.4 (3)	O4—C6—C5	116.2 (4)
C4—N2—Cu1^i^	121.1 (3)		
			
Cu1—O1—C1—O2	−173.2 (3)	N2—N1—C3—N3	−0.2 (5)
Cu1—O1—C1—C2	11.6 (7)	N2—N1—C3—C2	179.4 (4)
Cu1^i^—O3—C6—O4	168.2 (3)	N2—C4—C5—C6	41.1 (6)
Cu1^i^—O3—C6—C5	−11.0 (6)	N3—C4—C5—C6	−142.1 (4)
Cu1—N1—N2—Cu1^i^	24.3 (5)	C1—C2—C3—N1	26.4 (7)
Cu1—N1—N2—C4	−161.7 (3)	C1—C2—C3—N3	−154.0 (4)
Cu1—N1—C3—N3	162.8 (3)	C3—N1—N2—Cu1^i^	−173.6 (3)
Cu1—N1—C3—C2	−17.6 (7)	C3—N1—N2—C4	0.4 (5)
Cu1^i^—N2—C4—N3	174.4 (3)	C3—N3—C4—N2	0.2 (5)
Cu1^i^—N2—C4—C5	−8.4 (6)	C3—N3—C4—C5	−177.1 (4)
O1—C1—C2—C3	−21.3 (6)	C4—N3—C3—N1	0.0 (5)
O2—C1—C2—C3	163.0 (4)	C4—N3—C3—C2	−179.7 (4)
N1—N2—C4—N3	−0.4 (5)	C4—C5—C6—O3	−30.0 (6)
N1—N2—C4—C5	176.8 (4)	C4—C5—C6—O4	150.8 (4)

Symmetry code: (i) −*x*+1, −*y*+1, −*z*+1.

### Bis[µ-2,2'-(4H-1,2,4-triazole-3,5-diyl)diacetato]bis[diaquacopper(II)] dihydrate Hydrogen-bond geometry (Å, º)

**Table:**

*D*—H···*A*	*D*—H	H···*A*	*D*···*A*	*D*—H···*A*
O1*W*—H1*WA*···O4^ii^	0.87	1.91	2.762 (5)	165
O1*W*—H1*WB*···O3*W*	0.87	1.94	2.797 (5)	169
O2*W*—H2*WA*···O1*W*^iii^	0.85	2.03	2.880 (4)	170
O2*W*—H2*WB*···O2^iv^	0.87	1.89	2.744 (4)	167
N3—H3···O4^v^	0.88	1.81	2.670 (5)	165
C5—H5*B*···O3^v^	0.99	2.32	3.257 (6)	158

Symmetry codes: (ii) *x*+1/2, −*y*+3/2, *z*+1/2; (iii) *x*−1, *y*, *z*; (iv) −*x*+1/2, *y*−1/2, −*z*+3/2; (v) −*x*+1/2, *y*+1/2, −*z*+1/2.
