# Supplementary figures and images for: Synthesis, crystal structure and Hirshfeld surface analysis of a new copper(II) complex based on diethyl 2,2′-(4H-1,2,4-triazole-3,5-di­yl)di­acetate
Source: Acta Crystallogr E Crystallogr Commun. 2024 Aug 30;80(Pt 9):976–80. doi: 10.1107/S2056989024008259 (PMC11389672; doi:10.1107/S2056989024008259)

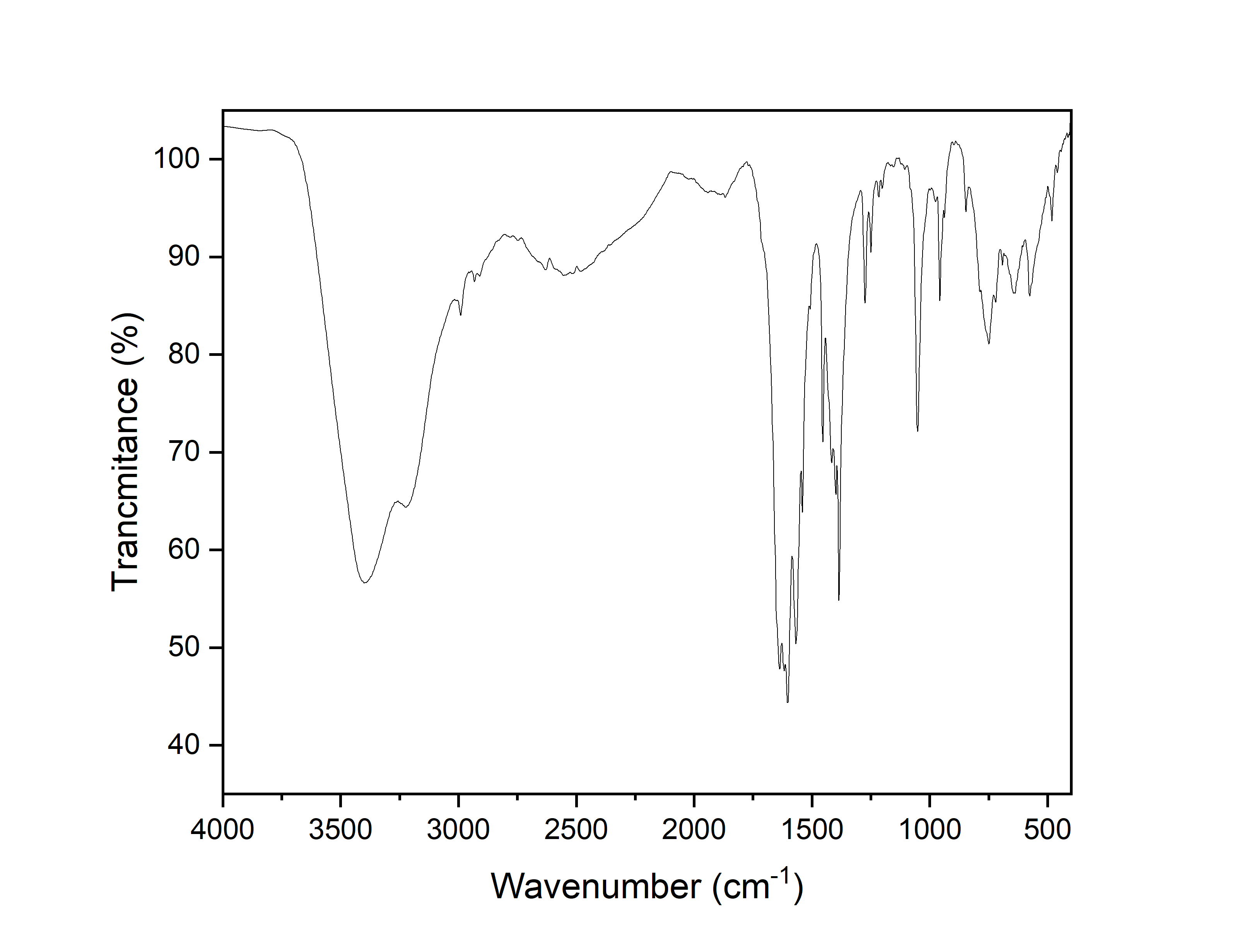

Supplement: Supplementary file 4 [file e-80-00976-sup4.jpg]

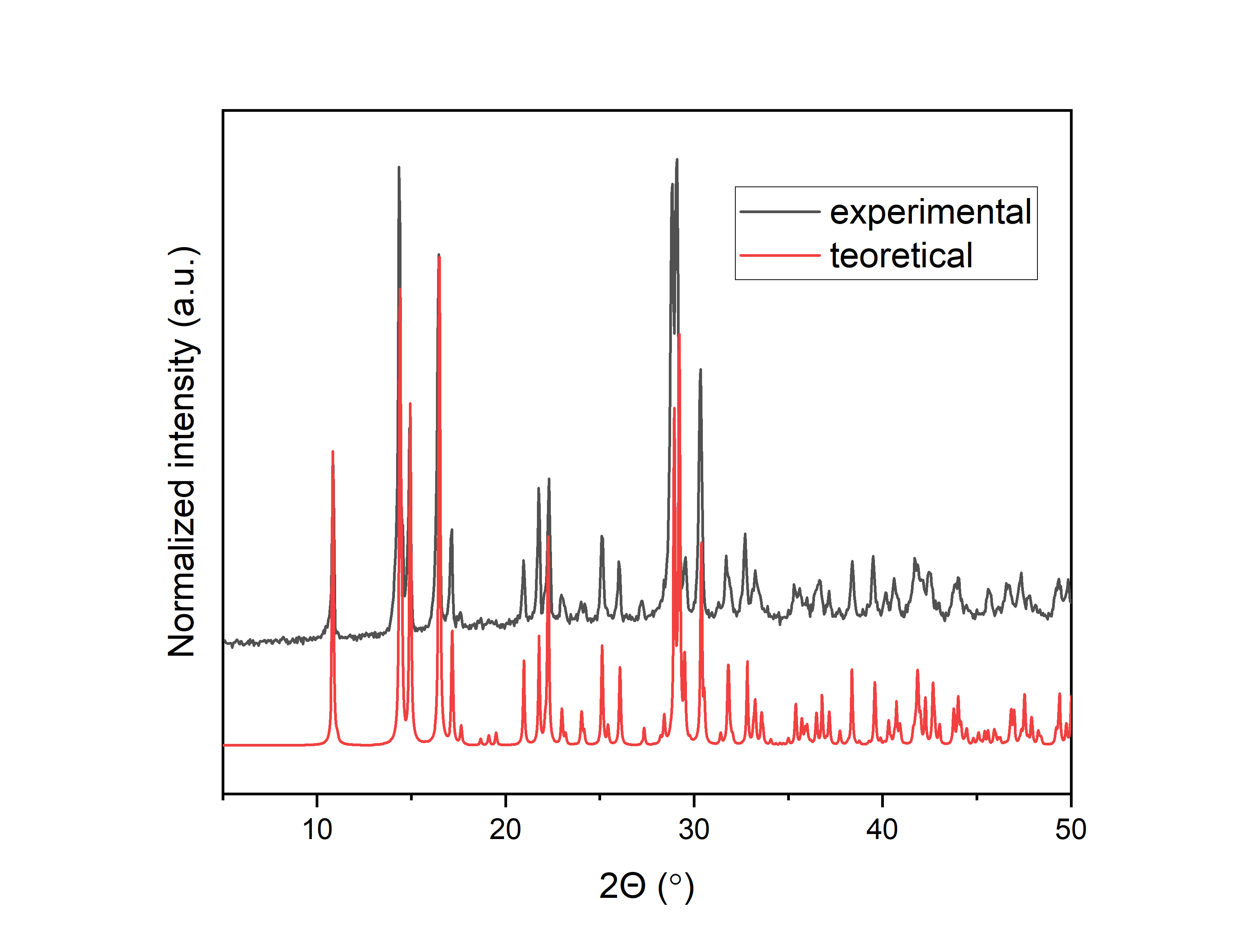

Supplement: Supplementary file 5 [file e-80-00976-sup5.jpg]
